# Inhibitory effects of three genuine resin glycosides from *Calystegia hederacea* on *in vitro* porcine lipase assay and *in silico* docking simulation analysis

**DOI:** 10.1007/s11418-026-02017-6

**Published:** 2026-03-09

**Authors:** Hirotaka Nishikawa, Masashi Hirano, Hideki Kinoshita, Kazunari Yoneda, Masateru Ono, Shin Yasuda

**Affiliations:** 1 Graduate School of Bioscience, Tokai University, Mashiki-Machi, Kamimashiki-Gun, Kumamoto, 861-2205 Japan; 2 Research Institute of Agriculture, Tokai University, Mashiki-Machi, Kamimashiki-Gun, Kumamoto, 861-2205 Japan

**Keywords:** Covolvulaceae, Pancreatic lipase, Calyhedin, Lipase inhibitor, Docking simulation

## Abstract

Resin glycosides are characteristically present in Convolvulaceae plants. We recently demonstrated that three genuine resin glycosides, muricatins V, VI, and IX, from the seeds of *Ipomoea muricata*, inhibit lipase. This study investigated whether the resin glycoside fraction (CHRG Fr.) obtained from the whole plants of *Calystegia hederacea* and three resin glycosides, calyhedins II (1), III (2), and VIII (3), from the rhizomes of this plant, can inhibit porcine lipase *in vitro*. The results indicated the inhibitory effects of CHRG Fr. (IC_50_; 33.1 µg/mL) and 1–3 (IC_50_; 4.5–23.7 µM). *In silico* molecular docking simulations revealed that 1–3 bind stably to the catalytic sites of porcine (PDB ID: 1ETH) and human (PDB ID: 1LPB) pancreatic lipases. Using data from 1 to 3 and three muricatins that we had previously determined, we observed a high correlation (R^2^ = 0.817, log_10_ (*a*; IC_50_ value) = 0.472799 x (*b*; docking score) + 5.9412, *n* = 6) between *in vitro* IC_50_ values and *in silico* binding scores for porcine lipase. By a leave-one-out cross-validation approach, we confirmed that this equation is a useful model with moderate predictive performance. There was also a high correlation (R^2^ = 0.928) in binding scores between porcine and human lipases. In conclusion, this study is the first to demonstrate that 1–3 from *C. hederacea* can inhibit porcine lipase *in vitro* and bind stably to lipase catalytic sites *in silico*. Therefore, it is suggested that resin glycosides from *C. hederacea* may serve as unique lipase inhibitors. (246 words)

**Graphical abstract:**

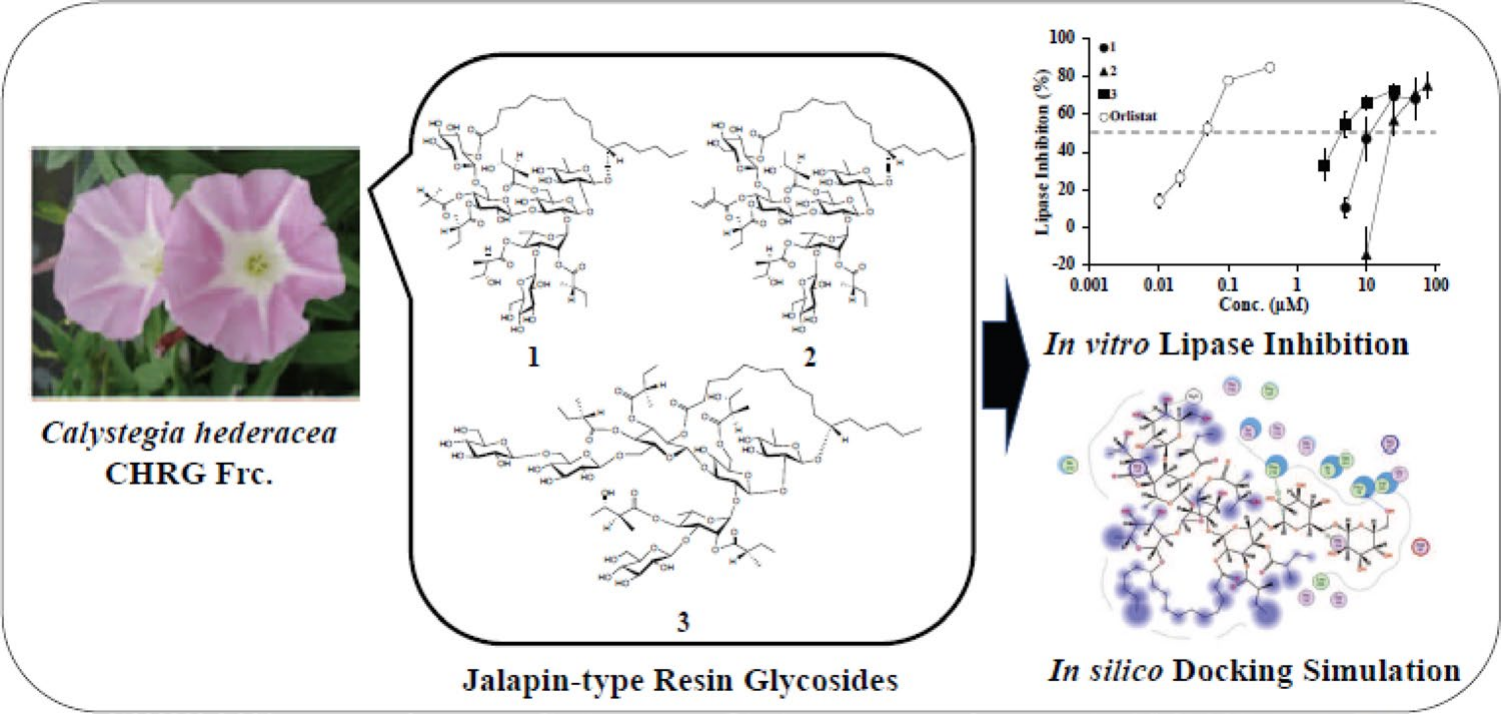

**Supplementary Information:**

The online version contains supplementary material available at 10.1007/s11418-026-02017-6.

## Introduction

Resin glycosides are characteristic constituents of Convolvulaceae plants and are considered active ingredients in purgative drugs, such as Pharbitidis Semen (the seeds of *Ipomoea nil* Choisy), Rhizoma Jalapae (the root of *Ipomoea purga* (Wender) Hayne) and Orizabae Jalap Tuber (the tuber of *I. orizabensis* (Pelletan) Ledanois) [1, 2]. Resin glycosides of sweet potatoes are also present in the white and sticky latex that emerges on the surface when the edible part is cut [3]. Chemically, resin glycosides are composed of glycosidic acids (oligoglycosides of hydroxyl fatty acids) as a core structure [1, 2]. Some of the hydroxyl groups in the sugar moiety are acylated by organic acids. Throughout our study of resin glycoside, we proposed that they can be classified into two types. The first type is the jalapin type, which contains a macrolactone structure. The second type is the convolvulin type, which contains an acylated glycosidic acid with a free carboxylic acid group of aglycone moiety. Due to their structural complexity and molecular weight of approximately 1,000–2,000, researchers have focused on isolating and determining the structure of resin glycoside, as well as investigating their potential biological activities.

*Calystegia hederacea* Wall. (Convolvulaceae), is a widely distributed perennial herbaceous vine native to India and East Asia. It has been traditionally used in medicine and all parts of the plant have been employed to treat disorders such as menstrual irregularities and gonorrhea [4]. We previously isolated and structurally determined several jalapin-type resin glycosides from this plant [5–10]. In those studies, we also reported some of their cytotoxic activity against HL-60 leukemia cells for antitumor purposes, as well as their anti-herpes activity against simplex virus type-1.

The multifunctional effects of several resin glycosides from various Convolvulaceae plants have been reported, including cytotoxic effects on cancer cells [11–13], antiviral activity [14–17], and anti-inflammatory properties [18, 19]. Additionally, there have been reports of inhibitory effects on digestive enzymes. For instance, *I. batatas* resin glycoside extract has been shown to inhibit lipase [20], and various resin glycosides from the morning glory family [21], as well as *I. alba* [22], have demonstrated α-glucosidase inhibition.

The pharmacological potential of various secondary metabolites found in herbal plants has recently been investigated for therapeutic applications in managing metabolic conditions. For instance, flavonoids, a type of polyphenol, act as bioactive enzyme modulators by inhibiting carbohydrate-hydrolyzing enzymes such as α-glucosidase and α-amylase. This contributes to their therapeutic potential in addressing metabolic disorders, particularly hyperglycemia and diabetes [23–25]. Pancreatic lipase breaks down lipids into free fatty acids and monoglycerides, playing a role in lipid digestion and subsequent absorption [26]. Therefore, modulating lipase activity is an important approach for regulating metabolism in cases of hyperlipidemia and obesity.

Given the increasing global prevalence of obesity and metabolic syndrome, identifying novel and safe lipase inhibitors from natural sources and developing effective lipid control strategies is a promising research approach [26]. Although synthetic lipase inhibitors, such as orlistat, are clinically effective, concerns have been raised about their adverse side effects, including gastrointestinal discomfort and impaired nutrient absorption [26]. Recently, we demonstrated the selective lipase inhibition of resin glycoside fraction from *I. muricata* and three genuine resin glycosides (muricatins V, VI and IX) as the compounds responsible for the first time [27]. However, the medicinal properties of resin glycoside, especially those from *Calystegia* plants, are not as well elucidated as those of well-known *Ipomoea* plants, particularly with regard to their inhibitory effects on digestive enzymes.

**Fig. 1:**
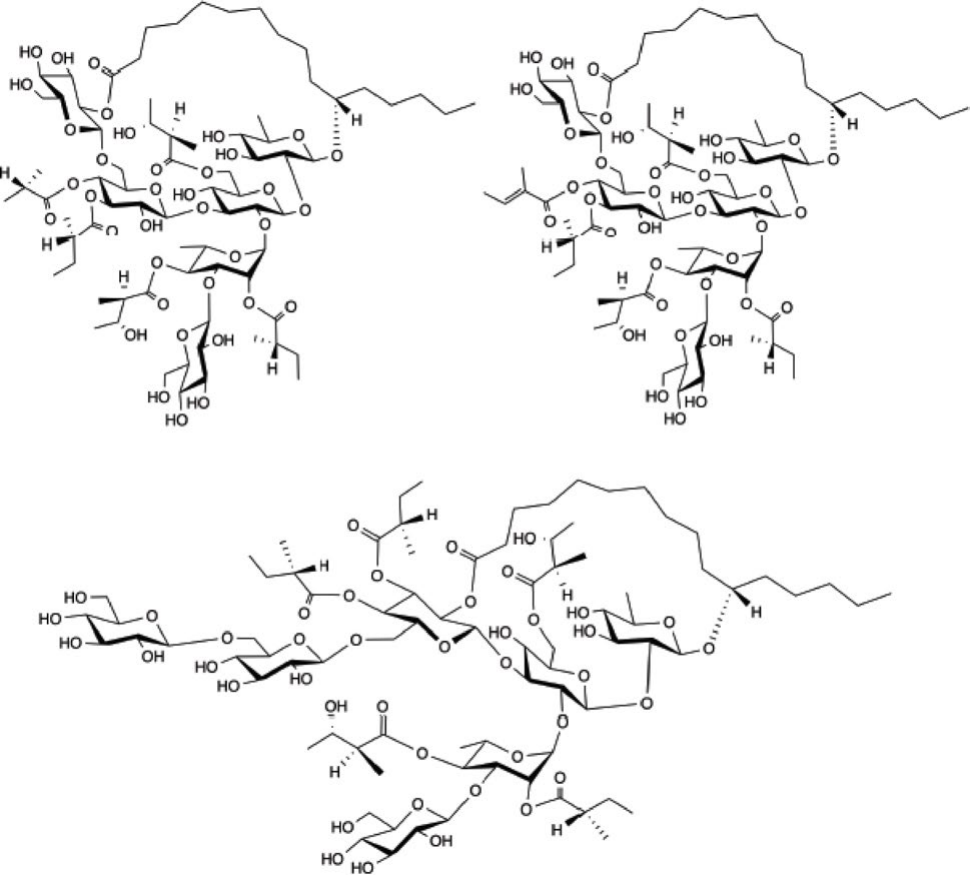
Chemical structures of calyhedins II (1), III (2), and VIII (3) used in this study. These three resin glycosides were previously isolated from *C. hederacea* [6, 7]

The current study aimed to investigate whether the resin glycoside fraction (CHRG Fr.) and three other genuine resin glycosides, namely calyhedins II (1), III (2), and VIII (3), previously obtained from *C. hederacea* (Fig. 1) could inhibit the porcine lipase *in vitro*. These three compounds were selected because sufficient amounts had been obtained for *in vitro* enzyme assays [6, 7]. Since porcine lipase was used in the *in vitro* inhibition assay, *in silico* molecular docking simulations were performed on the catalytic sites of porcine (PDB ID: 1ETH) and human (PDB ID: 1LPB) pancreatic lipases for compounds 1–3. We then performed correlation analyses on the data obtained *in vitro* and *in silico*.

## Results and discussion

**Fig. 2:**
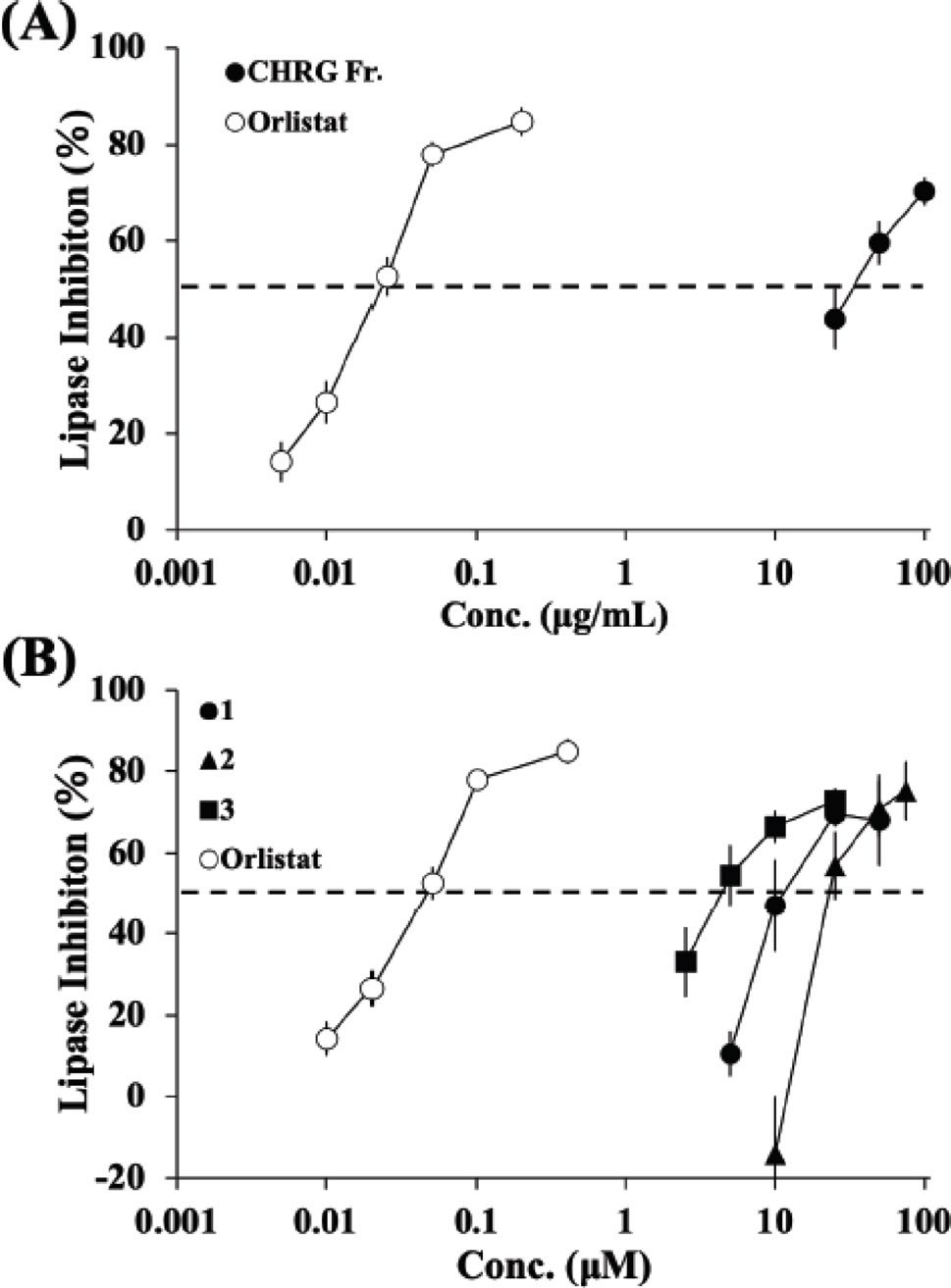
Inhibitory effects of CHRG Fr. (**A**) and 1–3 from *C. hederacea* (**B**) on an *in vitro* lipase assay. Data shown represent mean ± S.D. (*n* = 3). Porcine pancreatic lipase inhibition assay was performed using *p-*nitrophenyl laurate as substrate at 37 °C for 30 min. Orlistat was used as a positive control. CHRG Fr.; resin glycoside fraction from *C. hederacea*, 1; calyhedin II, 2; calyhedin III, 3; calyhedin VIII

First, we investigated whether CHRG Fr. could exhibit an inhibitory effect in a porcine lipase assay. The CHRG Fr. clearly inhibited pancreatic lipase, exhibiting an IC_50_ value of 33.1 µg/mL (Fig. 2A). The positive control, orlistat, showed an IC_50_ value of 0.0239 µg/mL (= 0.0481 µM) in this experiment. These results are comparable to those of other publications. Recently, resin glycoside extracts from *I. muricata* seeds (IC_50_; 21.0 µg/mL) and *I. nil* seeds (IC_50_; 34.3 µg/mL) were also found to inhibit lipase *in vitro* [27]. As a positive control, orlistat exhibited the expected inhibitory activity in our experimental setting. Reported IC_50_ values of orlistat are 0.0219 µg/mL (= 0.0441 µM) [27] and 0.0540 µg/mL (= 0.109 µM) [28], respectively, while the inhibiting capability may depend partly on the experimental conditions [29].

It is important to consider the kinetic validation of the current lipase inhibition assay. The IC_50_ values were determined using a linear equation between two concentration points falling on either side of 50% activity. To understand their relevance, we attempted to prepare dose-response curves with four-parameter logistic regression models in MATLAB software (see Supplementary Fig. S1). An additional calculation was performed using the log-logit linear transformation method as an inhibitory sigmoid model. Under these models, the calculated 95% confidence intervals for CHRG Fr. and orlistat were 16.6**–**35.6 µg/mL (Fig. S1A) and 0.0208**–**0.0292 µg/mL (= 0.0419–0.0589 µM) (Fig. S1B), respectively. Notably, the IC_50_ values of CHRG Fr. and orlistat were 21.7 µg/mL and 0.0255 µg/mL (= 0.0504 µM), respectively. These comparable results support the relevance of our initially determined IC_50_ values and the kinetic validation of this lipase inhibition assay.

Next, we investigated whether 1–3 could inhibit lipase using the same experimental setting. All three chemicals exhibited concentration-dependent inhibition (Fig. 2B). Their respective IC_50_ values are 12.6 µM, 23.7 µM, and 4.50 µM, respectively (Table 1). These IC_50_ values were initially determined using a linear equation. Using four-parameter logistic regression models in MATLAB software, the calculated 95% confidence intervals of 1–3 were 6.92–9.64 µM (Fig. S2A), 22.6–24.3 µM (Fig. S2B), and 2.29–3.40 µM (Fig. S2C), respectively. The respective IC_50_ values of 1–3 were 8.11 µM, 23.5 µM, and 2.83 µM. Due to the similar trend shown in 1–3 among both regression models, these closer results may partly support the relevance of our initial IC_50_ values. We recently demonstrated the lipase inhibition of three genuine jalapin-type resin glycoside (muricatins V, VI and IX) from *I. muricata*, with IC_50_ values ranging from 23.2 to 58.2 µM [27]. Thus, resin glycoside, particularly those from Convolvulaceae plants, are promising candidates for developing lipase inhibitors and for future health applications. However, more research is needed to determine if resin glycoside can play a role in human lipid-metabolizing enzymes and if they further modulate lipid digestion and absorption.

**Table 1:** IC_50_ values for 1–3 from *C. hederacea* in the lipase Inhibition assay

Compounds	IC_50_ (µM)
1	12.6 ± 3.6^a^
2	23.7 ± 1.2^b^
3	4.50 ± 0.94^c^
Orlistat	0.0481 ± 0.0039^c^

Data shown represent mean ± S.D. (*n* = 3). Porcine pancreatic lipase inhibition assay was performed using *p-*nitrophenyl laurate as substrate at 37 °C for 30 min. Tukey-Kramer’s test was performed for multiple comparisons, and values not sharing a common superscript letter are considered significantly different at *P* < 0.05. Orlistat was used as a positive control

**Fig. 3:**
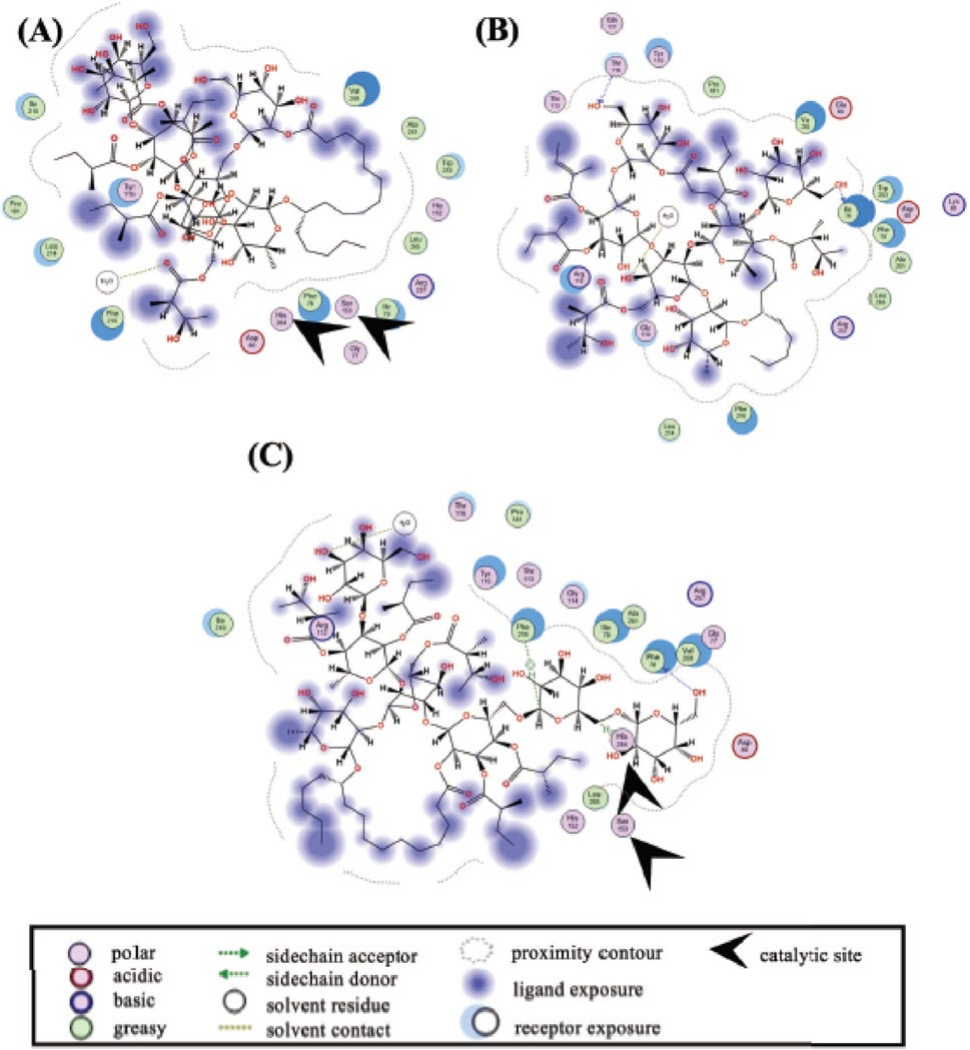
Two-dimensional docking pose of porcine pancreatic lipase (1ETH) with 1 (**A**), 2 (**B**), and 3 (**C**). Docking simulations of 1–3 and target protein at the potential binding site were individually performed using the MOE-Dock program

Porcine pancreatic lipase is often used to screen and characterize lipase inhibitors [30]. We also used this commercially available porcine lipase for *in vitro* assays of the resin glycosides. Next, we performed *in silico* Molecular Operating Environment (MOE) analyses using porcine (PDB ID: 1ETH) and human (PDB ID: 1LPB) lipases to predict their binding capabilities to the catalytic sites of these enzymes. As shown in Fig. 3, two-dimensional docking poses of porcine pancreatic lipase (1ETH) with 1–3 were obtained using MOE analysis. In this *in silico* analysis, simulating an optimized docking pose defines a negative and lower docking score as a parameter for lipase-ligand interactions and a highly stable binding state.

As shown in Table 2, comparable lower scores were observed for compounds 1 (–10.5883 kcal/mol, Fig. 3A), **2** (–10.0436 kcal/mol, Fig. 3B), and 3 (–10.6244 kcal/mol, Fig. 3C). In this experimental setting, the scores for the positive control, orlistat, and the substrate, *p*-nitrophenyl laurate, were previously determined to be − 9.0527 kcal/mol and − 7.6537 kcal/mol, respectively [27]. Three polar residues and seven hydrophobic residues were found to be common binding sites for 1–3, orlistat, and *p*-nitrophenyl laurate. Most of these compounds, except for 2, exhibit docking images close to the polar residues Ser153 and His264 of the catalytic site of porcine pancreatic lipase (1ETH) [31] (*see* Table 2; Fig. 3). It is unlikely that these compounds will interfere with the important catalytic site Asp177. These *in silico* data partially support the inhibitory mechanism(s) of 1–3 in porcine pancreatic lipase *in vitro* (*see* Fig. 2). Notably, 2 shows no docking pause at the three known binding sites, possibly due to the structural difference between the organic acid (e.g., a 2-methylbutyrate group on 1 vs. a tiglate group on 2), which may affect local hydrophilicity and binding affinity.

**Table 2:** *In silico* Docking simulation analysis and parameters of Porcine or human pancreatic lipase ligand interactions with 1–3 from *C. hederacea*

Compounds	Docking score (kcal/mol)	Polar residue	Hydrophobic residue	Hydrogen bond π-H stacking
Porcine pancreatic lipase (PDB ID: 1ETH)				
Common residue		Asp80, Tyr115, Arg257	Phe78, Ile79, Pro181, Phe216, Val260, Ala261, Leu265	
1	−10.5883	Gly77, His152, Ser153, His264	Ile210, Leu214, Trp253	
2	−10.0436	Lys81, Glu84, Arg112, Thr113, Gly114, Thr116, Gln117	Leu214, Trp253	Ile79, Thr116
3	−10.6244	Gly77, Arg112, Thr113, Gly114, Thr116, His152, Ser153, His264	Ile210	Phe78, Phe216, His264
Orlistat*	−9.0527	Gly77, His152, Ser153, His264	Ala179, Ile210, Leu214, Trp253	
*p*-Nitrophenyl laurate*	−7.6537	His152, Ser153, His264	Ala179, Ile210, Trp253	
Human pancreatic lipase (PDB ID: 1LPB)				
Common residue		Asp79, Tyr114, Arg256, His263	Phe77, Ile78, Pro180, Ile209, Phe215	
1	−11.2172	Arg111, Thr112, Gly113, Ser152, Gln244, Thr255, Asp257	Ala178, Leu213, Trp252, Phe258, Ala259, Leu264	
2	−10.9165	Ser152, Asn212, Thr255	Val210, Leu213, Ile245, Trp252, Phe258, Ala259, Ala260, Leu264	
3	−12.0292	Arg111, Thr112, Gly113, Thr115, Gln116, Thr255	Leu25, Val210, Leu213, Trp252, Ala259, Ala260	Phe77, Phe215
Orlistat*	−8.7424	Gly76, Arg111, His151, Ser152	Ala178, Trp252, Ala259, Leu264	
*p*-Nitrophenyl laurate*	−7.1490	Gly76, His151, Ser152	Leu264	

Docking simulations of 1–3 and target protein at the potential binding site were individually performed using the MOE-Dock program. *Data of orlistat, a lipase inhibitor, and *p-*nitrophenyl laurate, a substrate, are from our previous report [27]

**Fig. 4:**
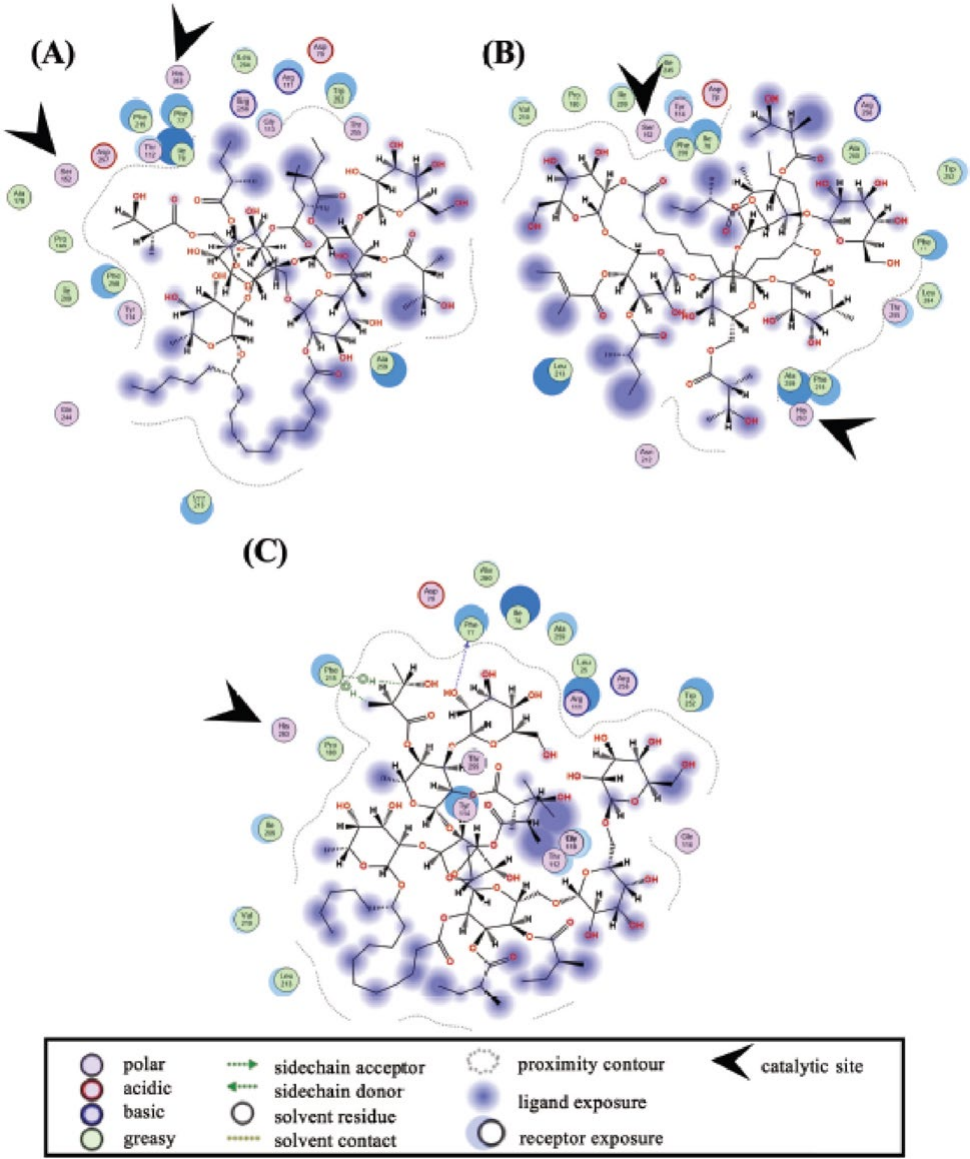
Two-dimensional docking pose of human pancreatic lipase (1LPB) with 1 (**A**), 2 (**B**), and 3 (**C**). Docking simulations of **1–3** and target protein at the potential binding site were individually performed using the MOE-Dock program

Figure 4 shows the two-dimensional docking pose of human pancreatic lipase (1LPB) with 1–3. Table 2 summarizes the results, which show lower and comparable scores for 1 (–11.2172 kcal/mol, Fig. 4A), **2** (–10.9165 kcal/mol, Fig. 4B), and 3 (–12.0292 kcal/mol, Fig. 4C). Previous studies have determined the values of orlistat (–8.7424 kcal/mol) and *p*-nitrophenyl laurate (–7.1490 kcal/mol) [27]. Four polar residues and five hydrophobic residues are common binding sites for 1–3, orlistat, and *p*-nitrophenyl laurate. Notably, our data for orlistat-lipase were comparable to data from another report [32] that employed MOE analysis. Most of these compounds exhibit simulated docking pauses near Ser152 and His263, the catalytic site of human pancreatic lipase [32]. With the exception of 3 (*see* Table 2; Fig. 4), interestingly, 1–3 exhibit lower values, indicating favorable binding scores, in both porcine and human pancreatic lipases. Therefore, it is suggested that 1–3 may act as inhibitors of human pancreatic lipase.

**Fig. 5:**
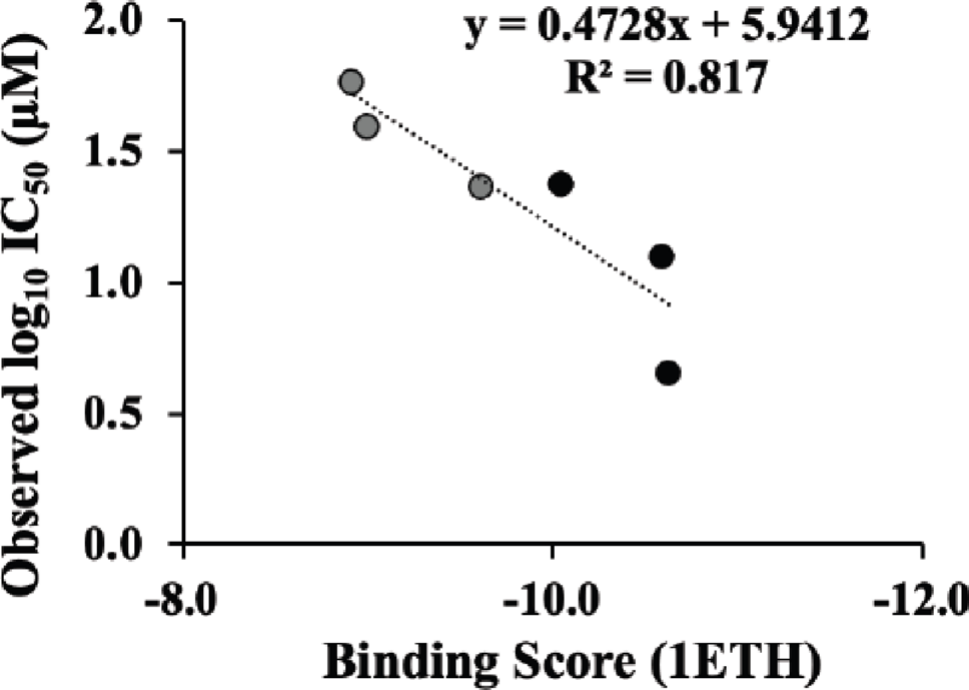
Relationship of the resin glycoside data with experimental IC_50_ values for *in vitro* porcine lipase inhibition and the *in silico* binding score (S-score) of resin glycoside to porcine lipase. The datasets of three resin glycosides (1–3) from *C. hederacea* (black-filled circles), which were tested in this study, and three others (muricatins V, VI, and IX) from *I. muricata* (gray-filled circles), which were reported in our previous study [27], were plotted in the figure. IC_50_ data indicates the mean value from the lipase inhibition assay (*n* = 3)

In this study, we pre-selected 1–3 as representative genuine resin glycosides because we had previously isolated and obtained sufficient quantities of them for structural determination and further *in vitro* assays [6, 7]. However, it is unclear whether resin glycosides with lower yields can inhibit lipase. To predict the inhibitory capability of various resin glycosides, we investigated the relationship between the *in vitro* lipase inhibitory activity and the *in silico* molecular docking scores of six jalapin-type resin glycosides. These included 1–3 from *C. hederacea* and also three additional muricatins (V, VI, and IX) from *I*. muricata, as those reported by us previously [27]. First, we performed a correlation analysis using the data from 1 to 3 and the three muricatins. Based on the linear logistic regression model, a high correlation (R^2^ = 0.817, log_10_ (*a*; IC_50_ value) = 0.472799 x (*b*; docking score) + 5.9412, *n* = 6) was observed between log_10_ IC_50_ values *in vitro* and binding scores *in silico* upon porcine lipase (Fig. 5). For instance, a correlation analysis with R^2^ = 0.519 (*n* = 9) has been demonstrated between experimentally determined K*i* values for P4-benzoxaborole derivatives against HCV NS3/4A protease *in vitro* and predicted docking scores *in silico* [33]. Another study showed an R^2^ value of 0.708 (*n* = 7) for flavone-type lipase inhibitors [34]. Notably, our model yielded relatively high correlation under the present settings, while R^2^ values may vary depending on target enzymes and compound structures.

It is important to evaluate the usefulness of this equation in estimating the inhibitory capacity of various resin glycosides based on their docking scores. We confirmed that this equation can be used with a simple leave-one-out cross-validation (LOOCV) approach. In this method, each observation in the entire dataset (i.e., experimental IC_50_ value and docking score of one sample) is used for validation estimation, while the remaining observations (IC_50_ values and docking scores of the other five samples) are used for the testing set. Table 3 shows that the predicted IC_50_ values of 1–3, muricatins V, VI, and IX were calculated to be 6.77**–**55.5 µM using both individual equation models and docking scores obtained *in silico*.

**Table 3:** LOOCV prediction of IC_50_ values of in vitro experimental lipase Inhibition using *in silico* Docking scores of 1–3 from *C. hederacea* and three other resin glycosides, namely muricatins V, VI, and IX from *I. muricata*

Resin glycoside	Experimental IC_50_ (µM)	Predicted IC_50_ (µM)	Regression equation (a = log_10_ IC_50_ value, b = docking score)	Fold Error**	Log_10_ Error***
**1**	12.6 ± 3.6	6.77	*a* = 0.548519 x *b* + 6.638308	1.86	−0.270
**2**	23.7 ± 1.2	14.1	*a* = 0.492346 x *b* + 6.095587	1.68	−0.224
**3**	4.50 ± 0.94	12.6	*a* = 0.341256 x *b* + 4.727330	2.81	0.448
Muricatin V*	58.2 ± 7.3	49.7	*a* = 0.451419 x *b* + 5.720576	1.17	−0.0682
Muricatin VI*	39.4 ± 6.0	55.5	*a* = 0.514761 x *b* + 6.377287	1.41	0.148
Muricatin IX*	23.2 ± 1.1	25.2	*a* = 0.475135 x *b* + 5.970318	1.09	0.0361

Experimental IC_50_ data, as observed, shown represent mean ± S.D. (*n* = 3). A linear logistic regression model was fitted in MATLAB software using both docking score (S-score) as the predictor and log_10_-converted IC_50_ values as the response. The predicted IC_50_ value of each compound was obtained by LOOCV, using a model trained on average data from remaining five other compounds (*n* = 5). LOOCV; leave-one-out cross validation

*The measured IC_50_ values of muricatins V, VI, and IX are from our previous report [27]

**Fold error: an error metric defined as the maximum ratio of Predicted/Observed or Observed/Pred (≥ 1)

***Log_10_ error: an error metric defined as log_10_ (Predicted) - log_10_ (Observed)

**Fig. 6:**
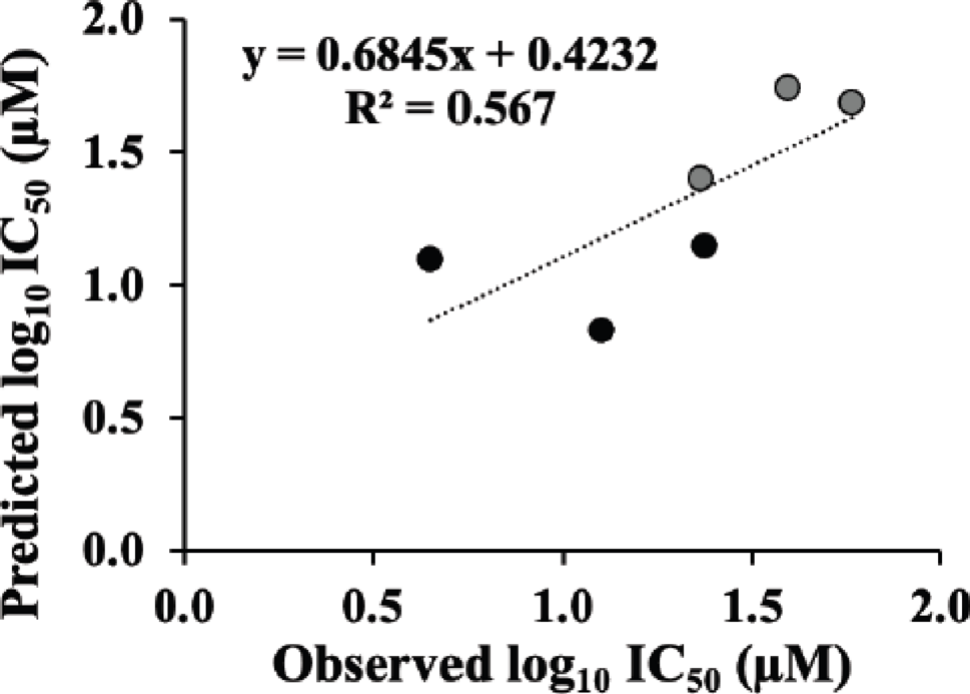
Scatterplots of predicted and experimental IC_50_ values in porcine pancreatic lipase inhibition for six resin glycosides. Experimental IC_50_ data represent mean value from independent biological experiments (*n* = 3). The predicted IC_50_ values were obtained by LOOCV using a simple linear regression model built between docking scores (S-score) and log_10_-transformed experimental IC_50_ values from five other compounds (*n* = 5). LOOCV; leave-one-out cross validation

The predictive performance of this model between the experimental and predicted IC₅₀ values was evaluated using several statistical metrics based on LOOCV. On prediction error metrics, fold error and Log_10_ error were 1.09**–**2.81 and − 0.0682**–**0.448, respectively. The calculated mean absolute error (MAE) and the root mean squared error (RMSE) of the model were 8.34 µM and 9.35 µM, respectively. After log_10_ transformation, the corresponding values were 0.199 and 0.242, indicating that the predicted IC_50_ values deviated from the experimental results by approximately 1.6- to 1.8-fold difference on average. The LOOCV-based determination coefficient (Q^2^_LOOCV_) was 0.543, indicating a moderate predictive performance. These results suggest that the docking score-based regression model provides a preliminary but reasonable estimation of the inhibitory activity of resin glycosides. The relationship between predicted and experimental IC_50_ values is simply visualized in Fig. 6 as a predicted-versus-observed scatter plot, on a logarithmic scale together with the regression line.

**Fig. 7:**
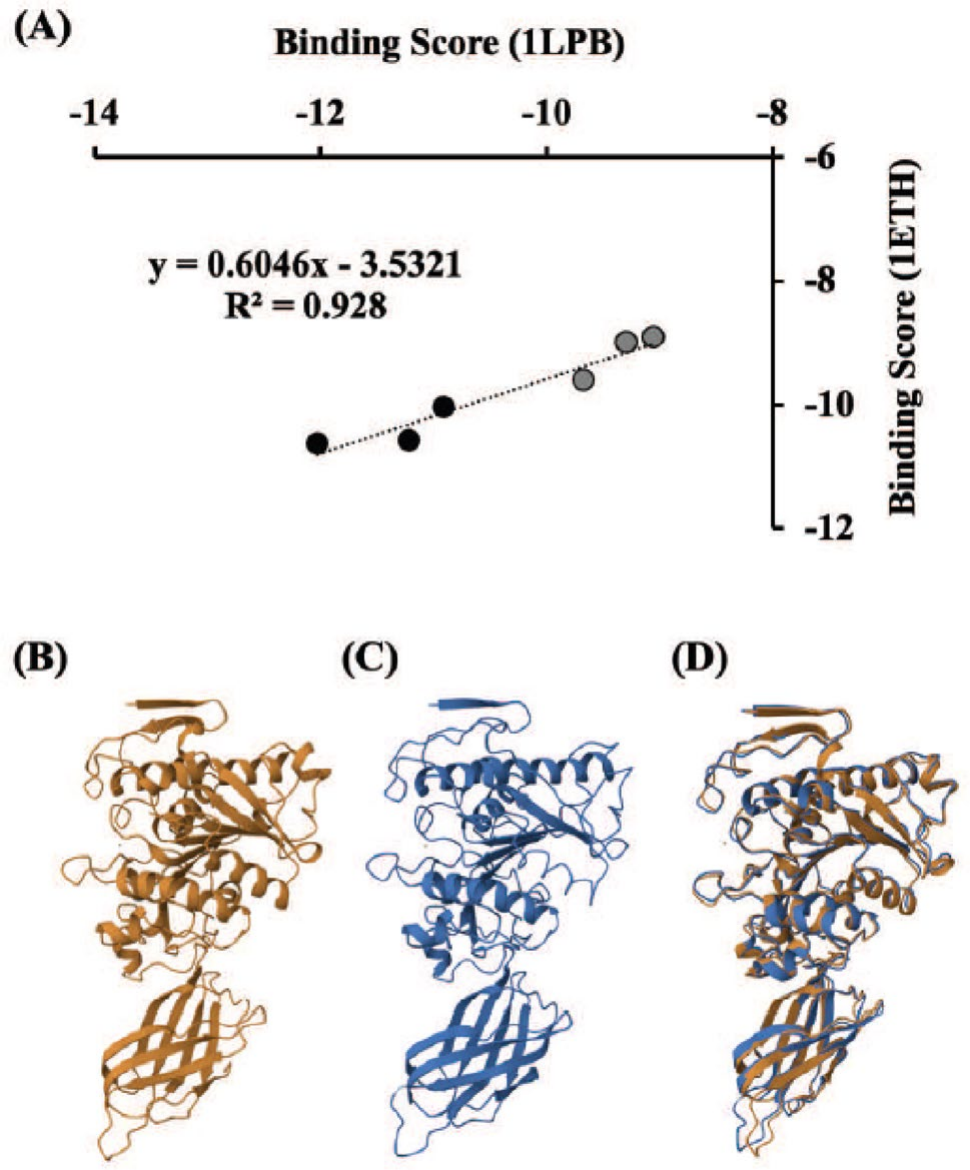
Correlation in binding scores between porcine (1ETH) and human (1LPB) lipases with six resin glycosides (**A**), structural comparison of porcine lipase (**B**) and human lipase (**C**), and their superimposed structures (**D**). In panel A, the *in silico* binding scores (S-score) of the resin glycosides to both porcine and human lipases were plotted. The datasets of three resin glycosides (**1–3**) from *C. hederacea* (black-filled circles) and three others (muricatins V, VI, and IX) from *I. muricata* (gray-filled circles) [27] were plotted. The protein structures of both enzymes (**B**, **C**) and their merged figure (**D**) were obtained from the Protein Data Bank

It should be noted that porcine pancreatic lipase is frequently used as a source of the enzyme when screening and characterizing lipase inhibitors, including synthetic drugs and phytochemicals from herbal medicines [30]. Interestingly, there was a high correlation (R^2^ = 0.928) in binding scores between porcine and human lipases with six resin glycosides (Fig. 7A). This strong correlation is consistent with previous structural analyses showing that porcine pancreatic lipase (PDB ID: 1ETH) and human pancreatic lipase (PDB ID: 1LPB) have highly conserved three-dimensional structure [35]. This suggests that the resin glycosides examined may inhibit human pancreatic lipase due to their high structural similarity, including their catalytic binding sites (approximately 86% similarity upon a structure similarity search in the Protein Data Bank). Figure 7 also shows a structural comparison of porcine (Fig. 7B) and human (Fig. 7C) lipases and their superimposed structures (Fig. 7D). However, the inhibition efficiency and mechanisms of the inhibitors in porcine and human pancreatic lipases may not always produce consistent results [36, 37]. Orlistat’s IC_50_ values differ between porcine and human pancreatic lipases, suggesting species-specific differences in enzyme sensitivity and inhibitor binding [36, 37]. Further experimental and computational validation using the human enzyme is needed.

Nevertheless, developing a useful predictive model for a variety of resin glycosides from porcine to human pancreatic lipases using *in silico* analysis is an important strategy. Our approach relies solely on docking scores as proxies for binding free energy, rather than incorporating multiple descriptors or machine learning optimization, which is typical of conventional quantitative structure-activity relationship (QSAR) studies. Molecular docking simulations are useful for qualitatively ranking ligand-protein affinity [38, 39]. To enhance the accuracy of quantitative predictions, additional QSAR models should also be considered.

These findings suggest that resin glycosides from *C. hederacea* could be used as potential lipase-inhibiting agents. However, it should be noted that the current study was limited to *in vitro* and *in silico*o evaluations. Since a chromogenic surrogate substrate was used for the porcine lipase assay, it is an interesting issue to determine whether the resin glycosides can directly inhibit triglyceride hydrolysis and affect lipid metabolism by both enzymes *in vitro* and *in vivo*. Docking scores provide an approximate estimate of binding affinity, which is influenced by model assumptions. The regression model derived from a limited set of resin glycosides requires further validation. Due to their large molecular size and amphiphilic nature, the intestinal absorption and systemic bioavailability of resin glycosides may be limited. This study provides preliminary evidence of lipase inhibition; however, *in vivo* efficacy remains unclear. Further studies focusing on the pharmacokinetic properties, solubility, and formulation stability of resin glycosides are needed to clarify their potential as lipase-targeting agents for therapeutic or functional applications. More work is needed to achieve this goal.

## Conclusion

In summary, we found that CHRG Fr. and three resin glycosides (1–3) from *C. hederacea* clearly inhibit porcine pancreatic lipase *in vitro*. *In silico* molecular docking simulations revealed that resin glycosides 1–3 bind stably to the catalytic sites of porcine and human pancreatic lipases. Due to the high correlation (R^2^ = 0.817) between the datasets of resin glycosides, which included IC_50_ values *in vitro* and binding scores *in silico*, the regression analysis equation was confirmed to be a useful predictive model with moderate predictive performance. It is suggested that resin glycosides from *C. hederacea* may serve as unique lipase-inhibiting agents. However, this study was limited to *in vitro* and *in silico* analyses. Further studies focusing on pharmacokinetics, bioavailability, *in vivo* efficacy, and formulation stability are needed to clarify their potential therapeutic applications. More research is necessary to develop a more reliable and broadly applicable predictive model for estimating the lipase inhibitory effects of various other resin glycosides.

## Experimental section

### Plant material

The whole plants (CHFUW2016) and rhizomes (CHFUR2012) of *C. hederacea* were collected in the Medical Plant Garden of Fukuoka University, Fukuoka, Japan, in September 2016 and October 2012, respectively, and identified by Dr. M. Okawa, as described previously [5, 6]. Each voucher specimen has been deposited at the laboratory of Natural Products Chemistry, School of Agriculture, Tokai University.

### Materials and reagents

CHRG fr. was obtained as a crude resin glycoside fr. in our previous study [5]. Briefly, the whole plant of *C. hederacea* was extracted with MeOH and partitioned between 70% MeOH and hexane. The 70% MeOH-soluble fr. was applied to Diaion HP20 column chromatography eluted with H_2_O–MeOH and acetone to obtain the CHRG fr. as a 90% MeOH eluate. 1–3 [6, 7] were previously obtained and used in this study. Orlistat and porcine pancreatic lipase were purchased from Tokyo Kasei Kogyo Co., Ltd. (Tokyo, Japan), and Nacalai Tesque, Inc. (Kyoto, Japan), respectively. All other chemicals were of the highest commercially available quality. The samples tested for the enzyme inhibition assays were prepared in dimethyl sulfoxide (DMSO), which did not affect the enzyme assay.

### Measurement of lipase Inhibition

Lipase inhibition was measured according to the method described in our previous report [27]. The sample solution 5 µL (in DMSO at a final concentration of 5%), Tris-borate-EDTA buffer solution 45 µL (89 mM, pH 8.3), and *p*-nitrophenyl laurate 25 µL (1 mg/mL in DMSO) were mixed in a well plate and preincubated at 37 °C for 5 min. Then, lipase 25 µL (1200 U/mL) in Tris-borate-EDTA buffer was centrifuged, preincubated at 37 °C for 5 min, and added to the assay mixture. The reaction proceeded at 37 °C for 30 min. Absorbance was measured at 400 nm using a microplate reader (SH-1000Lab, Corona Electric, Ibaraki, Japan). Orlistat in DMSO was used as a positive control.

### Determination of IC_50_ values and kinetic validation in lipase assay

In this study, the IC_50_ values were determined using a linear equation between two concentration points that fell on either side of 50% activity in Excel 2016 (Microsoft Co., Redmond, WA, USA). Subsequently, we prepared dose-response curves using four-parameter logistic regression models in MATLAB software (MathWorks, Natick, MA, USA) to determine 95% confidence intervals for IC_50_ values and kinetic validations in this lipase assay.

### *In silico* molecular Docking simulation

Structural data of the compounds as ligands for protein docking simulations was generated using ChemDraw software (Revvity Signals Software, Inc. Waltham, MA, USA). *In silico* molecular docking simulations were performed using MOE (v.2022.02) software (Chemical Computing Group ULC, Montreal, QC, Canada). Structural data for the positive control, orlistat (CAS: 96829-58-2), and the substrate, *p*-nitrophenyl laurate (CAS: 1956-11-2), were obtained using SciFinder CAS software (American Chemical Society). MOE expressed these ligands as a 2D electron diffraction model. Crystal structure data for porcine (PDB ID: 1ETH, https://www.rcsb.org/structure/1ETH) and human pancreatic lipase (PDB ID: 1LPB, https://www.rcsb.org/structure/1LPB) were obtained from the Protein Data Bank. Hydrogen atoms were added to these structures using the Protonate 3D module in MOE. The structures were optimized using energy minimization with AMBER10:EHT force field [40, 41].

Docking simulations of ligands and positive target proteins were performed using the MOE-Dock program. For porcine pancreatic lipase, the following amino acid residues were selected to define the potential binding site: His76, Gly77, Phe78, Asp80, Trp86, Tyr115, His152, Ser153, Leu154, Ala179, Glu180, Pro181, Phe216, Val260, Ala261, His264 and Leu265. These residues were formed into dummy atoms and brought closer to the catalytic sites Ser153, Asp177, and His264 [31]. For human pancreatic lipase, the following residues were selected for docking simulation, and their dummy atoms were brought closer to the catalytic sites Ser152, Asp176 and His263 [32]: His75, Gly76, Phe77, Ile78, Asp79, Tyr114, His151, Ser152, Ala178, Glu179, Pro180, Ile209, Phe215, Arg256, Ala260, His263, and Leu264. The scoring of each binding mode between the individual resin glycoside and lipase was based on the S-value (kcal/mol).

### Determination of predicted IC_50_ values

First, a simple linear regression analysis under Excel program was performed using data from six resin glycosides to examine the relationship between *in vitro* log_10_ IC_50_ values for porcine pancreatic lipase inhibition and *in silico* docking scores (S-scores) obtained from MOE analysis. Since IC_50_ values typically vary widely, the regression analysis used the base-10 logarithm of the IC_50_ values as the response variable and the S-score as the explanatory variable. The regression equation and coefficient of determination (R^2^) were obtained from the model fitted to all six compounds.

To assess the model’s robustness and predictive performance, leave-one-out cross-validation (LOOCV) was applied. For each iteration, one resin glycoside was removed from the dataset, and a regression equation was recalculated using the remaining five compounds. Then, the docking score of the omitted resin glycoside was substituted into the equation to obtain a cross-validated prediction of the log_10_ IC_50_ value in MATLAB software. This value was subsequently converted back to the IC_50_ scale. This procedure was repeated until each of the six compounds had been left out once.

Instead of “accuracy (%)”, predictive performance was evaluated using standard error metrics. Specifically, mean absolute error (MAE) and root mean square error (RMSE) were calculated based on the differences between the experimental and LOOCV-predicted IC_50_ values. To maintain consistency with model fitting, the MAE and RMSE were also computed on the log_10_-transformed IC_50_ scale. The predictive coefficient of determination (Q^2^_LOOCV_) was determined by comparing the sum of squared LOOCV prediction errors with the total variability of the observed logarithmic IC_50_ values.

### Statistical analysis

Values are expressed as the mean ± standard deviation from independent enzymatic/biological experiments (*n* = 3). Data analysis was performed using Statcel (OMS Co., Saitama, Japan), a statistical add-on software for Excel 2016 (Microsoft Co., Redmond, WA, USA). Tukey-Kramer test was performed for multiple comparisons, and values that did not share a common superscript were considered significantly different at *P* < 0.05.

## Supplementary Information

Below is the link to the electronic supplementary material.

Supplementary Material 1
