# Supplementary material for: Inhibitory effects of three genuine resin glycosides from Calystegia hederacea on in vitro porcine lipase assay and in silico docking simulation analysis
Source: J Nat Med. 2026 Mar 9;80(3):680–90. doi: 10.1007/s11418-026-02017-6 (PMC13186890; doi:10.1007/s11418-026-02017-6)
Supplement: Supplementary file 1 — Supplementary Material 1 [file 11418_2026_2017_MOESM1_ESM.docx]

***Supplementary Figures***

**Inhibitory effects of three genuine resin glycosides from *Calystegia hederacea* on *in vitro* porcine lipase assay and *in silico* docking simulation analysis**

Hirotaka Nishikawa · Masashi Hirano · Hideki Kinoshita · Kazunari Yoneda ·

Masateru Ono · Shin Yasuda

**List of Content**

Figure S1: Dose-response curves for CHRG Fr (A) and orlistat (B) using four-parameter logistic regression models in MATLAB software. Data shown represent mean ± SD (n=3).

Figure S2: Dose-response curves for **1**–**3** (A–C, respectively) using four-parameter logistic regression models in MATLAB software. Data shown represent mean ± SD (n=3).

**
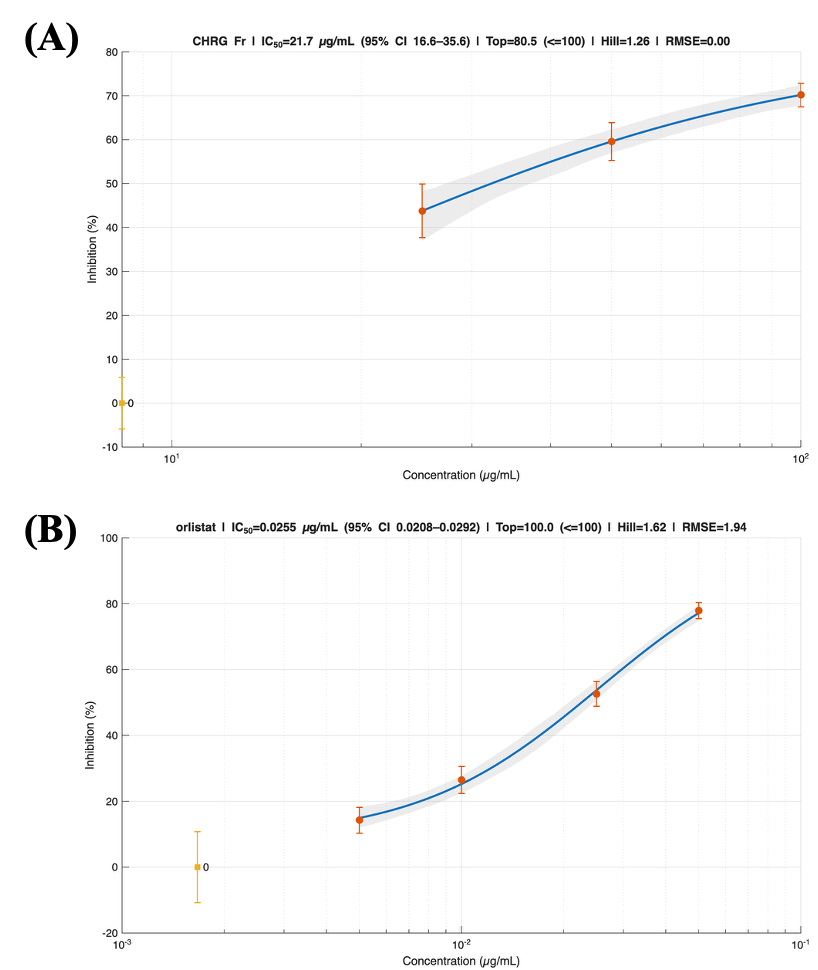
**

Figure S1: Dose-response curves for CHRG Fr (A) and orlistat (B) using four-parameter logistic regression models in MATLAB software. Data shown represent mean ± SD (n=3).


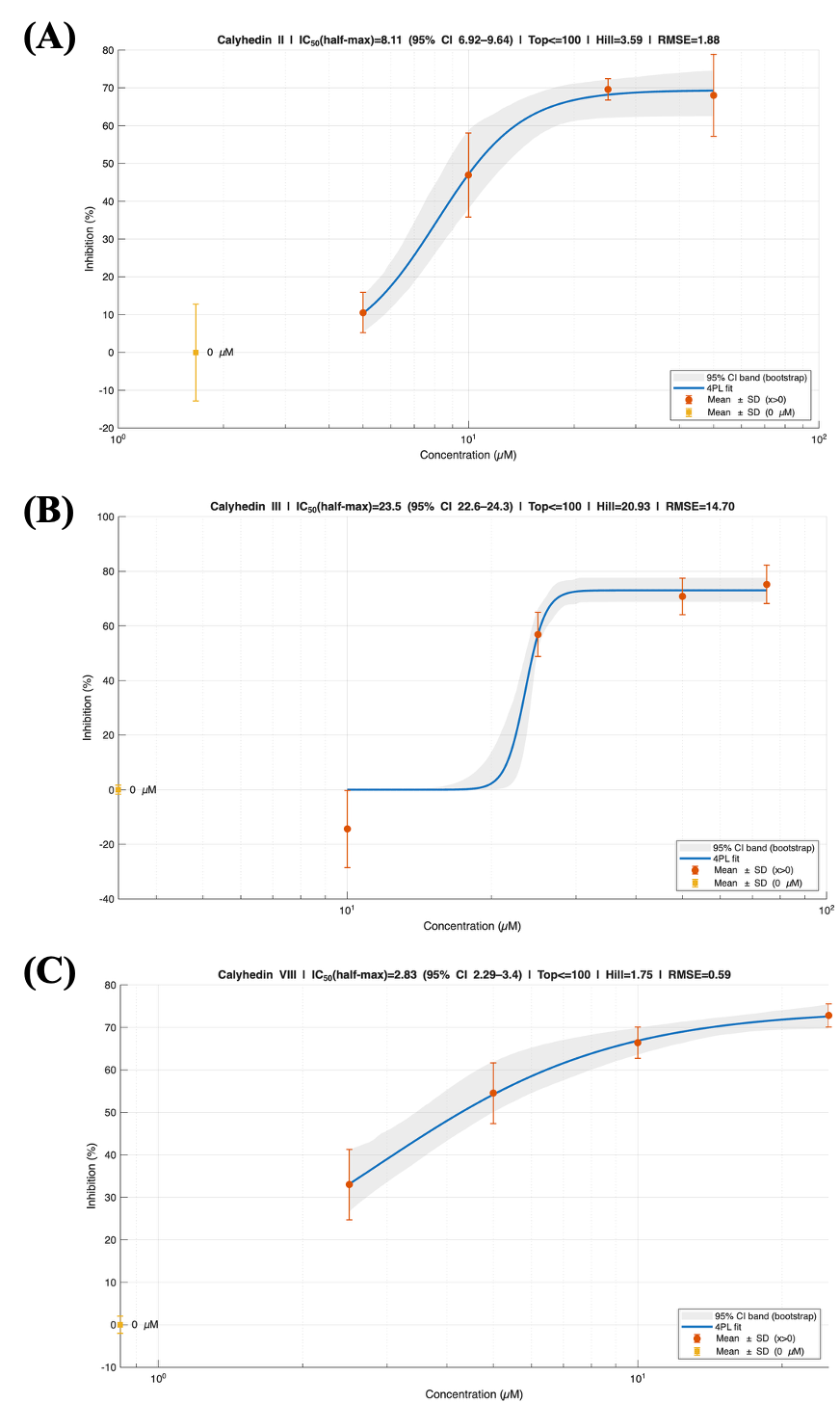


Figure S2: Dose-response curves for **1**–**3** (A–C, respectively) using four-parameter logistic regression models in MATLAB software. Data shown represent mean ± SD (n=3).
